# Ribosome profiling reveals translational regulation of mammalian cells in response to hypoxic stress

**DOI:** 10.1186/s12864-017-3996-8

**Published:** 2017-08-21

**Authors:** Zhiwen Jiang, Jiaqi Yang, Aimei Dai, Yuming Wang, Wei Li, Zhi Xie

**Affiliations:** 1 State Key Laboratory of Ophthalmology, Guangdong Provincial Key Lab of Ophthalmology and Visual Science, Zhongshan Ophthalmic Center, Sun Yat-sen University, Guangzhou, China; 2 Retinal Neurobiology Section, National Eye Institute, Bethesda, MD USA

**Keywords:** Hypoxia, Ribosome profiling, Translation efficiency, Loading ratio, Upstream open reading frame

## Abstract

**Background:**

Retinal pigment epithelium (RPE) cells transfer oxygen and nutrients from choroid to the neural retina. Reduced oxygen to RPE perturbs development and functions of blood vessels in retina. Previous efforts of genome-wide studies have been largely focused on transcriptional changes of cells in response to hypoxia. Recently developed ribosome profiling provides an opportunity to study genome-wide translational changes. To gain systemic insights into the transcriptional and translational regulation of cellular in response to hypoxic stress, we used simultaneous RNA sequencing and ribosome profiling on an RPE cells line, ARPE-19, under hypoxia condition.

**Results:**

Both HIF-1α and EPAS1 (HIF-2α) proteins were stabilized in ARPE-19 under hypoxic stress treatment at 1 h, 2 h and 4 h. Analysis of simultaneous RNA sequencing and ribosome profiling data showed genome-wide gene expression changes at both transcriptional and translational levels. Comparative analysis of ribosome profiling and RNA-seq data revealed that hypoxia induced changes of more genes at the translational than the transcriptional levels. Ribosomes densities at 5′ untranslated region (UTR) significantly increased under hypoxic stress. Interestingly, the increase in ribosome densities at 5′ UTR is positively correlated with the presence of upstream open reading frames (uORFs) in the 5′ UTR of mRNAs.

**Conclusion:**

Our results characterized translational profiles of mRNAs for a RPE cell line in response to hypoxia. In particular, uORFs play important roles in the regulation of translation efficiency by affecting ribosomes loading onto mRNAs. This study provides the first attempt to understand translational response of mammalian cells under hypoxic condition.

**Electronic supplementary material:**

The online version of this article (doi:10.1186/s12864-017-3996-8) contains supplementary material, which is available to authorized users.

## Background

Retinal pigment epithelium (RPE) cells transfer oxygen and nutrients from choroid to retina. Reduced oxygen to RPE perturbs development and functions of blood vessels in retina in various retinal pathologies such as age-related macular degeneration (AMD), proliferative diabetic retinopathy (PDR), retinopathy of prematurity (ROP) and glaucoma [1]. Previous studies showed that hypoxia inducible factors (HIFs) play a master role in the cellular response to hypoxia [2]. Using human RPE cells, it was found that HIF-1α expression increased under hypoxic stress condition [3, 4]. HIFs are a family of basic-helix-loop-helix transcription factors, which bind to HIF-responsive elements (HREs) in the promoter regions of hypoxia-activated target genes, modulating their gene expression [5]. One important HIFs’ target gene is vascular endothelial growth factor (VEGF). In rabbit RPE cells, VEGF expression was induced by hypoxic stress and reached to peak under hypoxic stress in six hours [6].

In addition to transcriptional regulation of HIFs, many previous studies also showed that HIFs regulate expression of their target genes at the translational level upon exposure to hypoxic stress. The translation of mRNA into protein is often divided into three main steps: initiation, elongation and termination [7]. Although the translational elongation process consumes approximately 99% of the energy needed for translation [7], translational initiation is generally regarded as the most crucial stage of controlling protein synthesis under hypoxic conditions [7]. EPAS1 (also known as HIF-2α) activates translational initiation of mRNAs containing RNA hypoxia responsive element (rHRE) in their 3′ UTRs to evade hypoxia-induced repression of protein synthesis via the EPAS1-RBM4-eIF4E2 complex [8]. However, there are certain differences between cellular responses to short-term and long-term hypoxic stress. Short-term hypoxia affects translational initiation via PERK-mediated eIF2α-phosphorylation, whereas prolonged hypoxia-induced translational change is regulated via down-regulation of mTOR activity mediated by REDD1 and AMPK, a process of PERK-independent eIF2α phosphorylation [9] and translational elongation [7]. Upon exposure to hypoxia, mTOR was inhibited via the tuberous sclerosis complex 1 and 2 (TSC1/2) in response to a shortage of energy production and REDD1, which is a transcriptional target of HIF-1α [10]. Under sustained hypoxia conditions, the inhibition of mTOR promotes increased binding of 4E–BP to eIF4E, resulting in an inhibition of cap-dependent translation [7].

Recently developed ribosome profiling based on the high-throughput sequencing of ribosome-protected mRNA footprints provides an opportunity to study genome-wide translational changes [7, 11]. For example, using ribosome profiling to assess gene expression levels of a neural cell line, PC12, during oxygen and glucose deprivation (OGD), it was found that more genes were affected at the translational level than the transcriptional level. Interestingly, OGD particularly led to increased ribosome density at 5′ leaders of mRNAs [12]. In another recent study, ribosome profiling was used to investigate translational regulation under hypoxic stress conditions in seedlings of *Arabidopsis thaliana*, illuminating prevalent and nuanced regulation of protein synthesis under hypoxia [13]. However, it is still largely unknown how mammalian cells are subject to genome-wide translational changes in response to hypoxia, which is important to understand many related human diseases such as cancer and neurodegenerative diseases [14].

In this study, we used simultaneous ribosome profiling (Ribo-seq) and mRNA sequencing (RNA-Seq) to establish quantitative transcriptional and translational responses of mammalian cells under hypoxic stress conditions using an RPE cells line, ARPE-19. Our study showed that both translational and transcriptional regulation participated in the cellular response of ARPE-19 to hypoxic stress, while more genes were affected at the translational level than the transcriptional level. We further demonstrated that the 5′ untranslated region (5′ UTR) helped genes resist hypoxia by regulating ribosome loading on mRNAs, and these regulatory roles mainly depend on the presence of uORFs located on the 5′ untranslated region. Altogether, our study characterized translational dynamics of ARPE-19 cells in response to hypoxic stress, revealing important roles that 5′ UTR and uORFs play in the translational regulation. To the best of our knowledge, this study provides the first attempt to understand translational response of mammalian cells under hypoxia.

## Results

### Hypoxia-induced factors in response to hypoxic stress in ARPE-19 cells

**Fig. 1:**
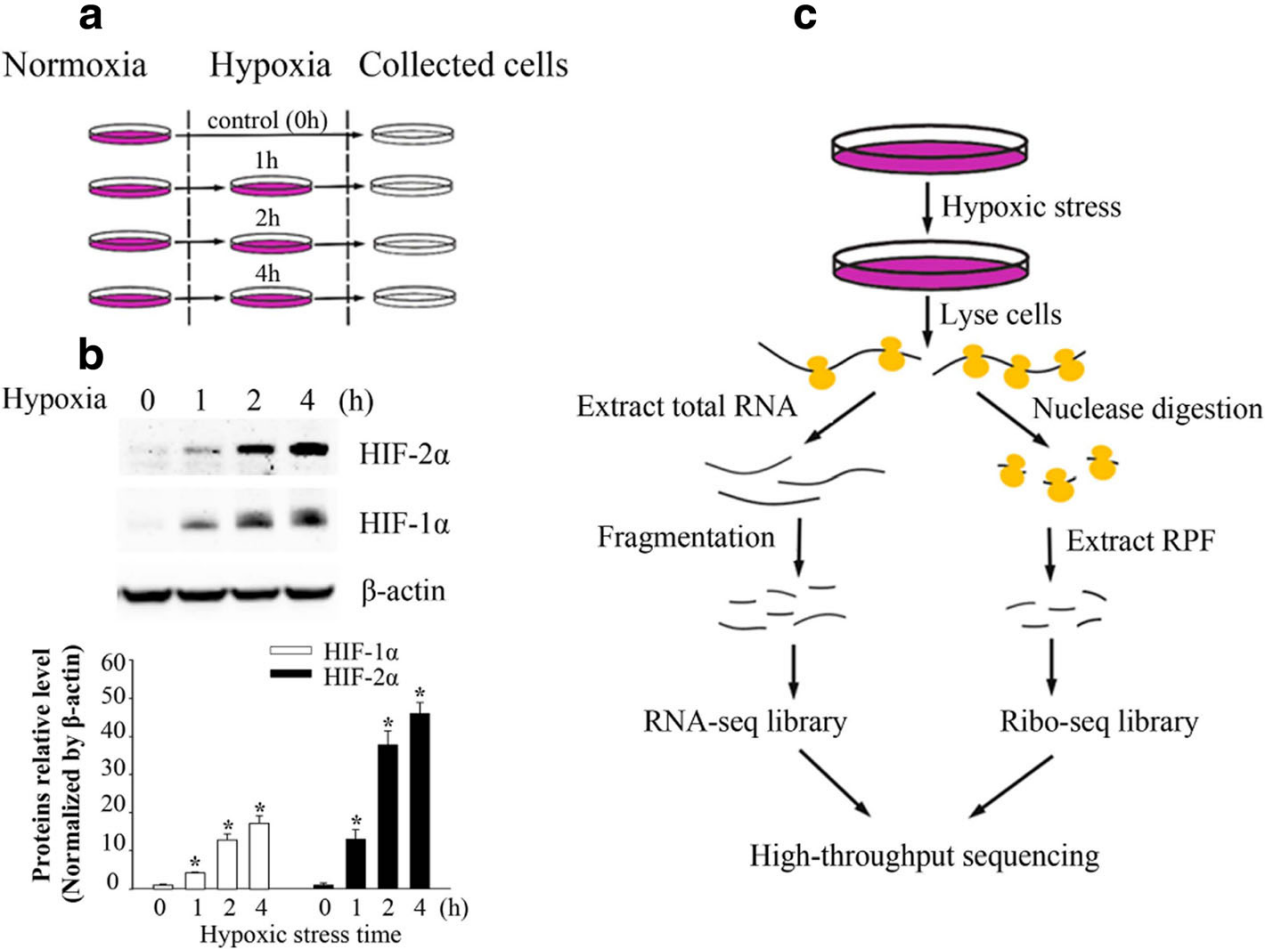
Simultaneous ribosome profiling/RNA-seq under hypoxic stress **a** Overview of experiments in ARPE-19 cells subject to hypoxic stress conditions. ARPE-19 cells were prepared in four separate dishes each containing 4 × 10^8^ cells. All time points greater than 0 h were cultured under 1% oxygen for hypoxic stress, and ARPE-19 cells cultured under 21% oxygen served as a control. Cells were harvested and analyzed using western blotting and high though-put sequencing technologies. **b** HIF-1α and HIF-2α (Epas1) protein levels in ARPE-19 cells were determined by western blotting. **c** Overall experimental design for simultaneous ribosome profiling and RNA-seq under hypoxic stress. ARPE-19 cells subject to hypoxic stress treatment for 0 h, 1 h, 2 h and 4 h. Polysome RNAs were extracted and digested with nuclease to construct the sequencing library for ribosome profiling, and total RNA was simultaneously extracted for RNA-sequencing (**c**)

We first cultured ARPE-19 cells under hypoxic stress (1% O_2_) for 1 h, 2 h and 4 h. In addition, we also maintained cells under the normoxic condition as a control (Fig. 1a). After cells were lysed, HIF-1α and Epas1 (HIF-2α) protein levels were determined by western blot assay. Compared with the normoxic condition, the HIF-1α protein level increased by approximately four-fold under the hypoxic condition. In addition, Epas1 expression was up-regulated approximately 12-fold when ARPE-19 cells were treated under hypoxic stress for 1 h, and high protein levels were sustained during prolonged hypoxic stress (Fig.1b). These results suggested that both HIF-1α and Epas1 were stabilized, demonstrating that ARPE-19 cells were disturbed by short-term hypoxic stress.

### High quality of ribosome profiling and RNA-seq data

**Fig. 2:**
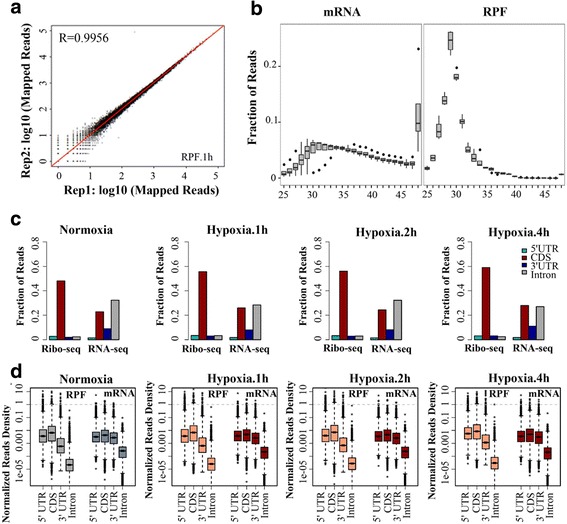
Global analysis of RNA-seq and ribosome profiling data **a** High reproducibility of the ribo-seq experiment under hypoxic stress: Pearson correlation coefficient is shown between two independent ribo-seq experiments under hypoxic stress for 1 h. **b** Length distribution of mRNA and RPF reads. Analysis of the insert size distribution of RPF reads and mRNA reads across all replicates and time points. The majority of RPF reads are 28 ~ 32 nt in length. **c** Reads distribution and (**d**) normalized read density of RPF reads and mRNA reads on genomic regions of 5’UTRs, CDSs, 3′ UTRs and introns of mRNAs are shown. RPF reads are presented on the left, and mRNA reads are presented on the right

We next performed simultaneous ribosome profiling and RNA-seq of ARPE-19 cells under hypoxic stress for 1, 2 and 4 h, as well as under the normoxic condition (0 h) (Fig. 1c). Ribosome profiling and RNA-seq quantitatively measured active ribosome protected fragments (RPF) and cellular mRNAs to unveil the dynamic translational and transcriptional changes. We generated adequate reads for further analysis (Additional file 1: Table S1). The almost perfect correlation between biological replicates (correlation coefficients >0.99) indicates that ribosome profiling experiments were highly reproducible (Fig. 2a). High reproducibility of ribosome profiling and RNA-seq experiments was also observed in ARPE-19 cells across different time points under hypoxic stress, as evidenced by correlation coefficients greater than 0.97 in all pairwise comparisons (Additional file 1: Figure S1). Analysis of sequencing read length indicated that the read lengths of ribosome profiling were approximately 28 ~ 32 nt (Fig. 2b), suggesting translated mRNA were protected by ribosomes from nuclease digestion [15, 16].

Ribosome profiling and RNA-seq experiments indicated distinctive features in the read densities of different genomic regions at translational and transcriptional levels, assessed by calculating the read numbers divided by the lengths of genomic regions and normalized by library sizes (Fig. 2c). Both ribosome profiling and RNA-seq experiments showed high read densities at coding sequence (CDS) and 5′ UTR regions. In contrast, ribosome profiling showed much lower read densities at 3′ UTR than that of RNA-seq, indicating termination of mRNA translation after stop codon (Fig. 2d). Overall, these results indicated that the ribosome profiling and RNA-seq experiments produced genome-wide transcriptional and translational data in high quality.

### Transcriptomic and translatomic changes upon exposure to hypoxia

**Fig. 3:**
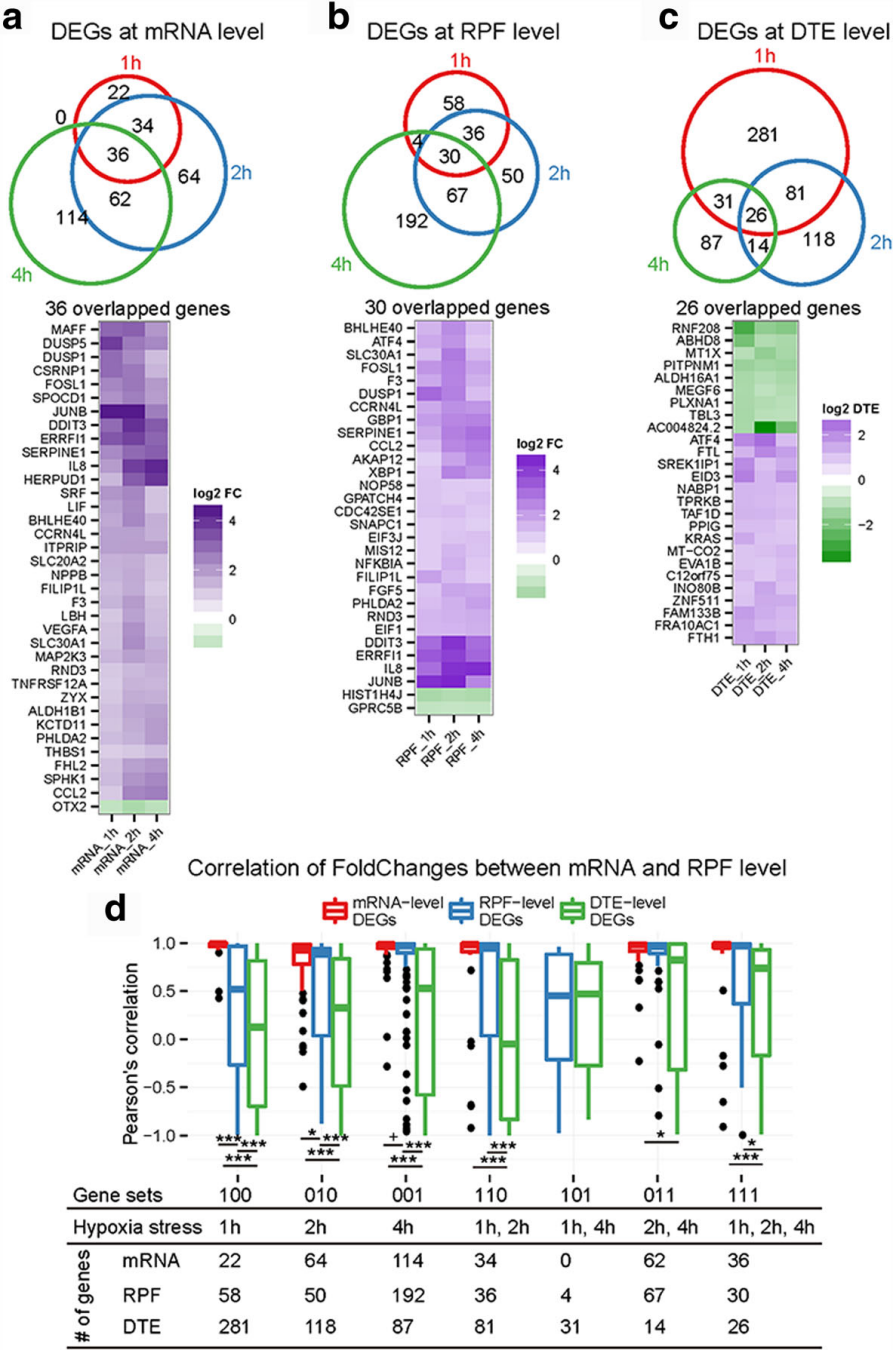
Correlation between transcriptional and translational changes. Differentially expressed genes (DEGs) under 1 h, 2 h and 4 h hypoxic stress at mRNA level **a** RPF level **b** and DTE level **c** Up panel: Venn diagram of overlapped genes across three time points; bottom panel: heatmap of DEGs presented at three time points. **d** Correlations between mRNA and RPF level changes. Pearson’s correlation were compared by gene set presented in the Venn diagrams. Statistical analysis was performed using Wilcox test: ^+^ means *p* < 0.1; * means *p* < 0.05; ** means *p* < 0.01; ** means *p* < 0.001

To assess genome-wide changes in transcription and translation upon exposure to hypoxic stress, we analyzed differentially expressed genes (DEGs) at the mRNA level and differentially translated genes (DTGs) at RPF levels. Both up-regulated and down-regulated genes were analyzed at three time points, and increased numbers of DEGs and DTGs were detected at the later time points (Additional file 1 Figure S2). At the RNA level, 92, 192 and 212 genes changed under hypoxia for 1 h, 2 h and 4 h, respectively (Additional file 2: Data 1). Gene ontology analysis revealed that significantly changed pathways associated with DEGs for all the three time points included “endoplasmic reticulum unfolded protein response”, “response to endoplasmic reticulum stress” and “response to hypoxia” (Additional file 3: Data 2). Analysis of ribosome profiling data revealed 128, 183 and 293 DTGs (fold change > 2 and counts >3.3) under hypoxic stress for 1 h, 2 h and 4 h, respectively (Additional file 2: Data 1). Gene ontology analysis showed that these differentially translated genes are associated with “response to endoplasmic reticulum stress”, “translation” and “regulation of transcription from RNA polymerase II promoter” (Additional file 4: Data 3). These results were consistent with previous findings showing that lack of oxygen supply led to the accumulation of unfolded proteins in the endoplasmic reticulum (ER stress) [7, 17] and as one of three distinct ER stress sensors, PERK (PKR-like ER kinase) was activated under hypoxic condition and mediated unfolded protein response [10, 18]. We also analyzed overlaps of all the differentially expressed genes at mRNA level, RPF level and translation efficiency level of ARPE-19 cells under hypoxic stress for 1 h, 2 h and 4 h. The Venn diagram shows 36 genes at mRNA level (Fig. 3a), 30 genes at RPF level (Fig. 3b) and 26 genes at translation efficiency level (Fig. 3c) were differentially changed in ARPE-19 cells in response to hypoxic stress for 1 h to 4 h. Upon hypoxic stress in ARPE19 cells, both HIF-1α target gene Vascular Endothelial Growth Factor A (VEGFA) and HIF-independent gene DDIT3 transcription level were increased (Fig. 3a), which is consistent with other mammalian cells upon exposure to hypoxia [19, 20]. Furthermore, transcription factor 4 (ATF4) translation was also induced (Fig. 3b, c), and this observation is also seen by others [21, 22]. To look further on the correlation of individual genes between transcriptional and translational changes, we then selected the overlapping and unique DEGs of mRNA, RPF and translation efficiency level across ARPE-19 cells under hypoxic stress for 1 h to 4 h. And then we separately calculated the pearson’s correlation coefficient of these genes between the fold change of mRNA and RPF level. As data shown in Fig. 3d, the DEGs from mRNA level, there was significant correlation between mRNA level and RPF level. However, the DEGs from RPF and DTE level exhibited a weaker correlation between mRNA level and RPF level changes than those from mRNA level. These results suggest the translation regulation may exist in ARPE-19 cells upon exposure to hypoxic stress.

We further performed Gene Set Enrichment Analysis (GSEA) of Kyoto Encyclopedia of Genes and Genomes (KEGG) pathway. At the transcriptional level, the number of affected pathways increased from 8 at 1 h to 17 at 2 h, and further increased to 23 at 4 h; Commonly enriched pathways among three time points include “MAPK signaling pathway” and “Toll-like receptor signaling pathway” (Additional file 1: Table S2). At the translational level, 41 pathways were enriched at 1 h and, 31 pathways were enriched at 2 h. At 4 h, 82 signal pathways were affected (Additional file 1: Table S2), where a large proportion of pathways were overlapped at transcriptional and translational levels (Additional file 1: Figure S3). A number of significantly regulated pathways during translational level were reported previously in response to hypoxic stress (Additional file 1: Table S3), such as “ubiquitin mediated proteolysis”, “oxidative phosphorylation” [12], “mTOR signaling pathway” [18] and “MAPK signaling pathway” [23]. These results indicated that translational and transcriptional regulations with hypoxic stress for ARPE-19 agreed with a general mechanism of other cell types.

We next performed relative translation efficiency analysis, which is defined as the ratio of normalized RPF density to normalized mRNA density. We identified 419, 239 and 158 of mRNAs (fold change > 2 and mean RPKM >5) with differential translation efficiency (DTE) for 1 h, 2 h and 4 h, respectively (Additional file 2: Data 1). We further carried out GSEA analysis of DTEs and identified a number of enriched pathways in response to hypoxia (Additional file 1: Table S4). In particular, The KEGG pathway “ribosome” was enriched at both 1 h and 2 h, suggesting translational regulation was significantly affected in ARPE-19 cells under short-term hypoxia [24].

### Dynamics of translational and transcriptional profiles

**Fig. 4:**
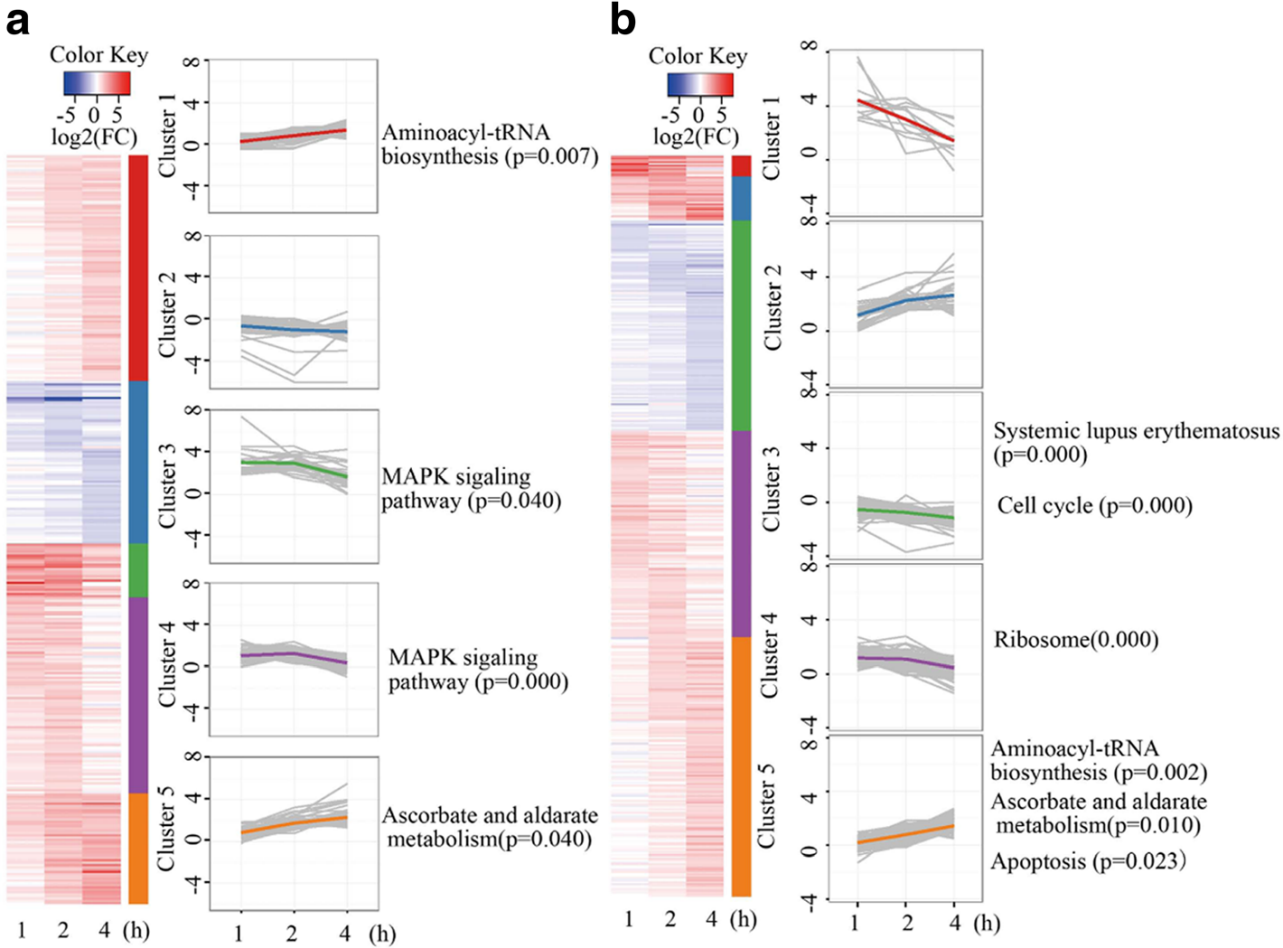
Cluster analysis of differential gene expression in ARPE-19 cells. ARPE-19 cells exposed to hypoxic stress conditions for 1 h, 2 h and 4 h. Cluster analysis of differential gene expression of ribosome profiling (**a**) and RNA sequencing (**b**)

We clustered genes based on the dynamic patterns of gene expression (RNA-seq) and translation (ribosome profiling) and performed functional annotation of these clustered genes. Clustering analysis of DEGs in RNA-seq revealed that the cluster 1 and the cluster 5 of gene expression at RNA level kept increasing, while the other clusters of genes expression decreased as hypoxia elongation. The significantly enriched pathways included aminoacyl-tRNA biosynthesis in the cluster 1, MAPK signaling pathway in the cluster 3 and the cluster 4, alanine and aspartate metabolism in the cluster 5 (Fig. 4a). On the other hand, clustering of the DTGs using Ribo-seq data revealed that the cluster 2 and the cluster 5 of genes were up-regulated, while the other clusters of genes were down-regulated as hypoxia prolongation. And the significantly enriched pathways include cell cycle in cluster 3, dysregulation of ribosome in the cluster 4 and up-regulation of aminoacyl-tRNA biosynthesis, alanine and aspartate metabolism, and apoptosis in the cluster 5 (Fig. 4b). During hypoxia, mitochondria increased generation of reactive oxygen species (ROS) [25], which activated mitogen-activated protein kinase (MAPK) [26]. Hypoxic conditions also lead to cell cycle arrest [27, 28] and apoptosis by a number of HIF-1-mediated and independent pathways [29, 30]. Ribosome and aminoacyl-tRNA biosynthesis pathways suggest that protein synthesis was affected under hypoxic stress [31, 32].

### Influence of upstream open reading frame on translational efficiency

**Fig. 5:**
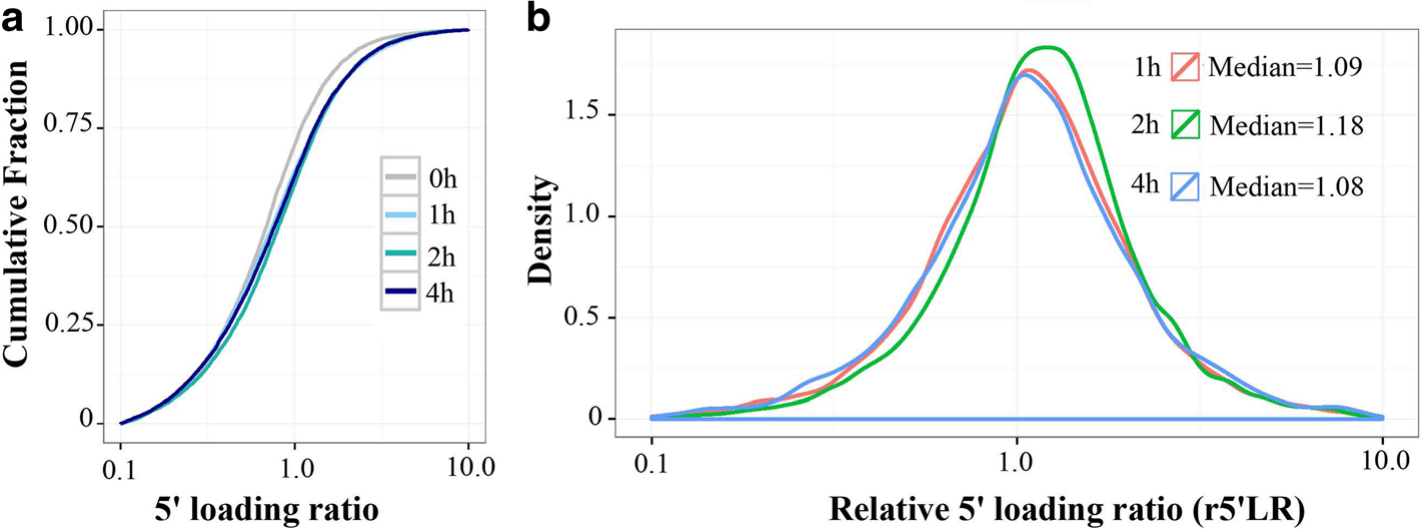
Ribosomes accumulate on the 5′-end of mRNAs in response to hypoxic stress. (**a**) Cumulative plots of the 5′ loading ratio of mRNAs are presented for ARPE-19 cells exposed to hypoxic stress conditions for 0 h (*gray*), 1 h (*light blue*), 2 h (*green*), and 4 h (*blue*). **b** Density plots of the relative 5′ loading ratio in ARPE-19 cells upon exposure to hypoxia. The median relative 5′ loading ratios are presented for 1 h (*red*), 2 h (*green*), and 4 h (*blue*)

Previous studies showed that ribosomes accumulated on ~200 nucleotides (nt) of CDS under heat shock stress [33], and this type of ribosome pausing could decrease the downstream ribosome footprint density and reduce peptide production in cells lacking EFP (Elongation factor P) [34]. To verify the 5′-end accumulation of ribosomes under hypoxic stress, we applied the 5′ loading ratio (5′ LR), which is defined as the ratio of ribosome footprint density on 5 ~ 65 codons to that on the remaining downstream CDS. Compared with 5′ LR distribution under normoxic condition, no significant differences in 5′ LR values of hypoxic stress were found for 1 h, 2 h and 4 h (Fig.5a). We further defined the r5′ LR as the ratio of 5′ LR under hypoxic stress to that under normoxic conditions. The median r5′ LR values increased from 1.09 to 1.18 as hypoxic stress treatment increased from 1 h to 2 h, and then dropped to 1.08 under hypoxic stress to 4 h (Fig.5b). The increase in 5′ LR values under hypoxic stress was apparent in most individual mRNAs examined, suggesting a global change in the translational machinery. Furthermore, the median of the r5′ LR values increased from 1 h to 2 h but decreased as hypoxic stress exposure time increased, indicating that translational processes likely aid in recovery as the hypoxic stress exposure time increases.

Translational regulation elements in the 5′ UTR of mRNA, including uORFs, internal ribosome entry sites (IRESs) and 5′ terminal oligo pyramiding tract (5′ TOP), play important roles in translation regulation [35]. To illustrate their roles in response to hypoxic stress, all coding genes were divided into two groups based on the presence of these regulation elements in the 5’ UTR of mRNAs. We first observed that genes containing uORFs had increased r5’ LR upon exposure to hypoxic stress at 1 h (*p* = 0.037, Wilcox test) and 2 h (*p* = 9.107e-05, Wilcox test) but not at 4 h (*p* = 0.252, Wilcox test). Translation efficiency of these genes containing uORFs also increased under hypoxic stress at 1 h (*p* = 0.87*10^−3^, Wilcox test), 2 h (*p* = 0.001, Wilcox test) and 4 h (*p* = 0.070, Wilcox test).

To further experimentally validate regulatory role of uORFs, we selected four genes containing uORFs in the 5’UTR of mRNA and cloned the uORF or no-uORF into pEZX-GA02 empty vector encoding secreted Gaussia Luciferase (GLuc) and Alkaline Phosphatase (SEAP) (Figs. 7a and b). Each of the reporter construct was transiently transfected into ARPE-19 cells and the GLuc activities were determined as the indicators of mRNA translation together with SEAP activities as transfection efficiency control. We observed that all the uORFs tested exhibited significantly higher GLuc activities in response to hypoxia for 2 h compared to the GLuc activities under normoxic condition. And all the no-uORFs tested reduced GLuc activities under hypoxic condition. Moreover, we also observed that all the uORFs tested reduced GLuc activities under normoxic and hypoxic conditions (Fig. 7c–f). These experimental results together with the global analysis indicated that the 5’UTR containing uORF took a translational advantage of mRNAs in response to hypoxia. The uORF of Erythropoietin (EPO) represses translation of the main ORF in ARPE-19 cells as in other proliferating cells under normal condition, and the repression of the main ORF of EPO translation was not observed under hypoxic stress [36]. This is consistent with some previous findings that uORFs can significantly reduce the translation efficiency of downstream ORF in unstressed conditions [37], while mutation of uORF partly rescued genes translation efficiency [38, 39]. Furthermore, genes with uORFs in their transcripts can promote an increase in translation efficiency of the main ORF in response to stress conditions in favor of them to evade global repression of translation [13, 40]. Internal ribosome entry site (IRES) elements were identified as functional cis-elements enable the cap-independent translation nearly 30 years ago within the 5’ untranslated region of picornavirus RNAs [41]. We also found that genes with IRES had increased r5’ LR under hypoxic stress at 1 h (*p* = 0.003619, Wilcox test), 2 h (*p* = 5.879e-05, Wilcox test) and 4 h (*p* = 0.007481, Wilcox test); however, translation efficiency was not affected upon exposure to hypoxic stress at 1 h (*p* = 0.4233, Wilcox test), 2 h (*p* = 0.3965, Wilcox test) and 4 h (*p* = 0.5452, Wilcox test). It should be noted that we used a common database (UTRdb) to identify transcripts carrying IRES elements [42, 43]. as IRES elements are elements of the secondary structure of RNA and only a few IRESes’ structure are available [44], they are notoriously hard to predict. Therefore, an RNA element was established as a functional IRES requires a number of carefully executed experiments with specific controls [45].

**Fig. 6:**
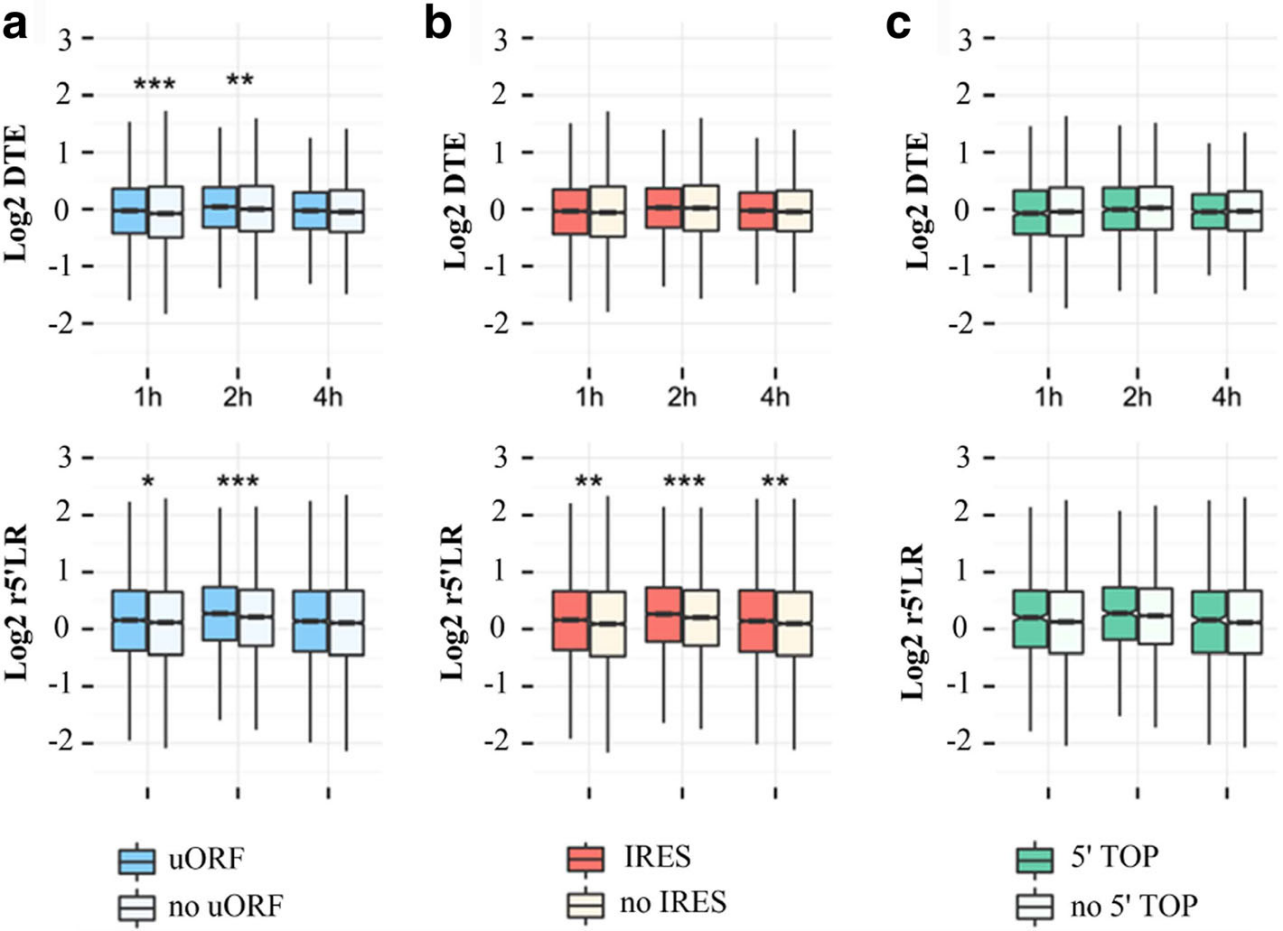
uORFs help to resist hypoxic stress. Genes were divided into two groups based on the presence of upstream open reading frames (uORFs), internal ribosome entry site (IRES) or 5′ terminal oligo pyramiding tract (5′ TOP). The effects of the uORF (A), IRES (B) and 5′ TOP on the 5′ loading ratio and translation efficiency were analyzed

In addition, we did not found that genes with 5’ TOP sequences exhibited significant advantages of mRNA upon exposure to hypoxic stress (*p* > 0.1, Wilcox test) (Fig. 6). These results implied that uORFs involved in the gene translational regulation responded to short-term hypoxic stress by increasing the ribosome loading ratio and the translation efficiency of cellular genes.

## Discussion

Under hypoxic conditions, translational control provides immediate and effective changes in protein levels compared with transcriptional regulation [10, 46]. Using ribosome profiling coupled with RNA sequencing, we explored gene expression of ARPE-19 upon exposure to hypoxia at the transcriptional and translational levels. Although both transcriptional and translational regulation of genes was found in response to hypoxic stress, more signaling pathways were affected at the translational level than the transcriptional level. With increased exposure to hypoxia, an increasing number of signaling pathways overlapped between the transcriptional and translational levels. Similarly, as previously reported, when the neural cell line PC12 was exposed to 1 h of oxygen and glucose deprivation (OGD), only 100 genes were significantly altered at the transcriptional level, whereas approximately 3000 genes were altered at the translational level [12]. Our results suggested that the translation of mRNAs was primarily altered by short-term hypoxic stress.

When considering ribosomes loading onto 5′-end of ORFs in mRNAs, we identified a subset of genes with an increased 5′ loading ratio under hypoxic stress. This finding may be due to multiple regulatory mechanisms, such as ribosome transient (reversible) pause, irreversible stalling, and acceleration or premature termination of translation elongation. Although the precise mechanisms underlying the increased 5′ LR under hypoxic stress remain unknown, the up-regulation of the 5′ LR may affect translational efficiency [33]. In many cases, 5′-leader sequence features of the corresponding mRNAs, such as regulatory upstream open reading frames (uORFs) and/or internal ribosome entry sites (IRESs), could help to evade global repression of translation in response to stress conditions [35]. The uORFs, IRES and 5’TOP in the 5’UTR of cellular mRNAs are involved in the regulation of gene translation upon exposure to hypoxia. Our results showed that with the exception of 5’TOP, both uORFs and IRES affected the r5’LR of genes; however, only uORFs induced gene translation efficiency. uORFs are emerging as important mediators of transcript-specific translational control of their downstream coding sequences [47]. Typically, uORFs lead to a significant reduction of the translation efficiency of the main downstream ORF under unstressed conditions [48], however, when eIF2 was phosphorylated and global translation was consequently inhibited under stress conditions, the presence of uORF(s) in a transcript promotes an increase in the corresponding protein levels [40]. Using ribosome-profiling technology to investigate translational regulation under normoxia and sublethal hypoxic stress conditions in seedlings of *Arabidopsis thaliana*, Juntawong et al. reported that uORFs are translated and that these uORFs act as a barrier to mORF (main ORF) translation under conditions of normoxia [13]. Moreover, they also observed that translational inhibition by a uORF was reduced during hypoxia and that ribosome occupancy in the intercistronic and mORF regions of uORF mRNAs increased and the translation efficiency of numerous mRNAs with uORFs were increased in response to hypoxia [13]. In eukaryotes, the scanning model of translation initiation involves the 43S complex, which includes a 40S ribosomal subunit that scans downstream along the 5′-untranslated region in a 5’to 3′ direction until it encounters the first AUG triplet [49]. When the upstream ORF is translated, the 40S ribosomal subunit exhibits leaky scanning or reinitiates at the downstream ORF to reinstate protein synthesis [50, 51]. It was thought that shorter uORFs and longer distances between the stop codon of the uORF and the next start AUG contributes to the resumption of leaky scanning and re-initiation. Some translational factors remain connected to the ribosome after translation of a short uORF, and the 40S ribosomal subunit has sufficient time to bind with the corresponding translation initiation factors to facilitate the re-initiation process [47]. A good example of this type of regulation is activating transcription factor 4 (ATF4), which promotes transcriptional up-regulation of specific target genes in response to cellular stress [52]. ATF4 expression is regulated by two uORFs at the translational level, with the second uORF overlapping the AUG of the ATF4 coding sequence, although in a different reading frame. Under normal conditions, the translation of the ATF4 coding sequence is prevented due to the second uORF overlapping with the main coding sequence. However, when eIF2a is phosphorylated under cellular stress conditions, the ternary complex is limited and the scanning preinitiation complex decreases initiation from the second uORF, increasing protein expression of mRNAs with the correct arrangement of uORFs [40].

## Conclusions

To sum up, we applied ribosome profiling and mRNA-seq experiments demonstrated that translational regulation was primarily affected in ARPE-19 cells under hypoxic stress. Although global gene translation was inhibited, the presence of a uORF promoted specific gene translation. These results suggest that uORFs play significant roles in the regulation of gene expression in response to hypoxia.

## Methods

### Cell culture

ARPE-19 cells were purchased from ATCC and were maintained in DMEM/F12 medium supplemented with fetal bovine serum and 1:2000 Mycoplasma OUT™ in a humidified incubator at 5% CO_2_ and 37 °C. Before experiments, the ARPE-19 cell line was confirmed with STR profiling analysis and mycoplasma determination. ARPE-19 cells were seeded in 10-cm Corning dishes and maintained in the adherent state for up to 2 days before hypoxic stress. Medium was replaced with fresh growth medium, and then ARPE-19 cells were cultured in the hypoxia incubator room (equilibrated with 95% N2 and 1% O2) at 37 °C. Cells were then incubated in hypoxia conditions for 1 h, 2 h and 4 h. Control cells were incubated in normoxic conditions (21% O2, 5% CO2).

### Western blott assay

For western blot analyses, cells were lysed in RIPA buffer (50 mM Tris-HCl, pH 7.2, 150 mM NaCl, 1% NP40, 0.1% SDS, 0.5% DOC, 1 mM PMSF, and supplemented with proteinase inhibitor cocktail). For normalization, β-actin signals were used, and the ImageJ program was used for quantitative data analysis. Experiments were performed in triplicate.

### Library preparation

After hypoxic stress, ARPE-19 cells were subsequently washed with cold PBS supplemented with 100 μg/ml cycloheximide followed by the addition of lysis buffer. Ribosome protected fragments were generated by treatment with 3 μl of ARTseq nuclease. A ribosome footprint (RPF) library and total RNA library were generated with the ARTseqTM Ribosome Profiling Kit based on the manufacturer’s protocol (RPHMR12126, Epicentre). RPF and mRNA libraries were sequenced on an Illumina HiSeq 2500 sequencer with TruSeq SBS Kit v3 for single-end 50 cycles (SE50) run type.

### Data processing, mapping and differential gene expression analysis

The adaptors of mRNA-seq and ribosome profiling reads were trimmed by cutadapt (v 1.4.2, cut adapt -a AGATCGGAAGAGCACACGTCTGAACTCCAGTCA -m 25). The tRNA and rRNA were removed by Bowtie (v1.0.1, −l 20) [53]. The tDNA reference sequences were obtained from the Genomic tRNA Database (http://gtrnadb.ucsc.edu/download/GtRNAdb/) [54], and the rDNA reference sequences were obtained from the abundant sequences of iGenomes (https://support.illumina.com/sequencing/sequencing_software/igenome.html). The reads were mapped to the human genome (Ensembl, GRCh37) using Tophat (v2.0.11) [55]. Raw counts of protein-coding genes, including mitochondrial genes, were quantified by HTSeq (v0.6.1p2) [56]. Then, RPKMs (reads per kilobase of transcript per million reads mapped) were calculated with edgeR [57], using total mapped reads as normalization factors. RPKMs were used as gene expression levels to calculate differentially expressed genes at both the transcriptional (mRNA) and translational (RPF) levels by comparing expression levels under hypoxia conditions to normoxic conditions. Genes with an absolute value of log2 fold-change ≥1 and an averaged logarithmic RPKM of corresponding conditions ≥3.3 were recognized as differentially expressed. Then, differentially expressed genes were clustered based on exposure time, and functional annotation was performed on clusters using a hypergeometric test in the R package “piano” [58].

### Calculating read density on genomic regions

For annotation of canonical isoforms, the longest CDSs of transcripts were used, and the 5′ UTR, 3′ UTR and introns were extracted according to the exon of the gene (Ensembl, GRCh37). Then, lengths of genomic regions were estimated according to the annotations. Raw read counts on these four regions were quantified by HTSeq (v0.6.1p2) [56]. Read density was calculated as raw read counts divided by the length of the corresponding genomic regions.

### Calculation of 5′ loading ratios

RPF reads were allocated to a specific A-site location as described previously [59]. Depending on the length of the reads, the position of +14 offset from the 5′ end of the alignment was defined as the A-site for the reads that are 25 ~ 28 nucleotides (nt) long; +15 is the A-site for the reads that are 29–30 nt long; +16 is the A-site for the reads that are 31–33 nt long; and +17 for the reads that are 34–35 nt long. Reads with a length of less than 25 nt or greater than 35 nt were excluded. The footprint density of a gene was measured based on its canonical isoform with the longest CDS. The ribosome 5′ loading ratio (5’LR) of a gene was defined as the ratio of the footprint density between codons 6 and 65 (bases 16 to 195) of the ORF to the density along the remaining downstream positions in the canonical ORF [33]. The relative 5′ loading ratio (r5′ LR) was calculated by the ratio of the 5’LR under hypoxic conditions to that of normoxic conditions.

### Translational regulation roles of elements in the 5′ UTR

Information about 5′ UTR elements, including uORF, IRES and 5′ TOP, was downloaded from UTRdb (http://utrdb.ba.itb.cnr.it/). Then, r5’ LR and DTE were compared between genes with regulatory elements and those without.

### Plasmid construction and luciferase activity measurement

**Fig 7.**
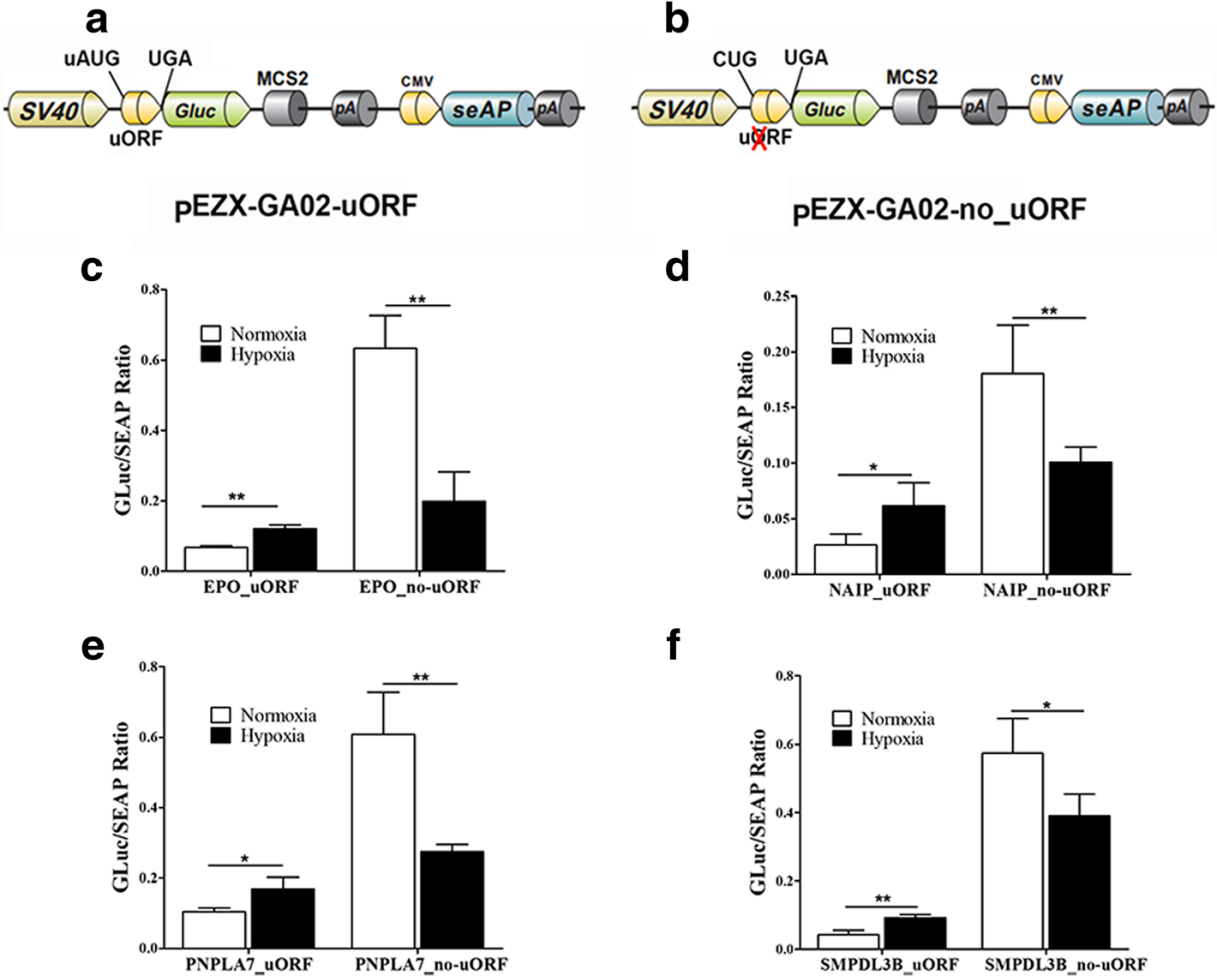
uORFs regulated translation of the downstream main ORF. (**a**, **b**) Schematic representation of reporter constructs. **a** pEZX-GA02_uORF contained the human EPO, NAIP, PNPLA7 and SMPDL3B uORFs with the intact initiation (uAUG) and termination (UGA) codons cloned into the empty vector (pEZX-GA02). **b** In the pEZX-GA02_no-uORF construct, the uORF initiation codon was mutated (AUG → CUG; the cross represents the nonfunctional uORF). **c**, **d**, **e**, **f** EPO, NAIP, PNPLA7 and SMPDL3B uORFs and no-uORFs affected Gluc activity representing translational efficiency (relative Gluc activity/SEAP activity) under nomorxic and hypoxic conditions. Average values of at least three independent experiments are shown. The error bars indicate standard deviation. Statistical analysis was performed using Student’s t-test: * means *p* < 0.05 and ** means *p* < 0.01

The plasmid pEZX-GA02 encoding secreted Gaussia Luciferase (GLuc) and Alkaline Phosphatase (SEAP) was used to determine luciferase activity with the dual-luciferase reporter assay system (GeneCopoeia). Synthesized fragments corresponding to the human uORF or no-uORF (Additional file 1: Table S5) were cloned into the pEZX-GA02 empty vector before Gluc coding sequence (Fig. 7a and b). Then pEZX-GA02_uORF or pEZX-GA02_no-uORF was transfected in ARPE-19 cells using Lipofectamine™ 3000 Reagent (Thermo Fisher Scientific). The luciferase activities were measured at 2 h post hypoxic stress (1% oxygen). The FB12 Luminometer (Berthold) was used to measure the luciferase activities using secrete-pair™ dual luminescence assay kit (GeneCopoeia) according to the manufacturers’ protocols. The ratio is luminescence intensities of the GLuc over SEAP, and each value was derived from at least three independent experiments.

## Additional files

Additional file 1: Figure S1. Reproducibility of ribo-seq and RNA-seq experiments. **Figure S2.** The number of differentially expressed genes increased as exposure to hypoxia increased. **Figure S3.** Dynamic translational regulation in ARPE-19 cells upon exposure to hypoxia. **Table S1.** Overview of ribosome profiling and RNA sequencing data. **Table S2.** Pathway enrichment of differentially expressed genes. **Table S3.** Pathway enrichment of differentially translated genes. **Table S4.** Pathway enrichment of genes with differential translation efficiency. **Table S5.** Synthesized uORFs/no-uORFs sequence. (DOCX 1503 kb)

Additional file 2: Data 1. Differentially expressed and translated genes and genes with differential translation efficiency. (XLSX 28 kb)

Additional file 3: Data 2. Biological process enrichment of differentially expressed genes. (XLSX 21 kb)

Additional file 4: Data 3. Biological process enrichment of differentially translated genes. (XLSX 13 kb)

## Abbreviations

3′ UTR 3′: Untranslated region
5′ LR 5′: Loading ratio
5′ TOP 5′: Terminal oligo pyramiding tract
5′ UTR: 5′ untranslated region
AMD: Age-related macular degeneration
CDS: Coding sequence
DEGs: Differentially expressed genes
DTE: Differential translation efficiency
DTGs: Differentially translated genes
EFP: Elongation factor P
GSEA: Gene Set Enrichment Analysis
HIFs: Hypoxia inducible factors
HREs: HIF-responsive elements
IRESs: Internal ribosome entry sites
KEGG: Kyoto Encyclopedia of Genes and Genomes
MAPK: Mitogen-activated protein kinase
OGD: Oxygen and glucose deprivation
PDR: Proliferative diabetic retinopathy
rHRE: RNA hypoxia responsive element
Ribo-seq: Ribosome profiling
RNA-Seq: mRNA sequencing
ROP: Retinopathy of prematurity
ROS: Reactive oxygen species
RPE: Retinal pigment epithelium
uORFs: Upstream open reading frames
VEGF: Vascular endothelial growth factor
