# Supplementary material for: Ribosome profiling reveals translational regulation of mammalian cells in response to hypoxic stress
Source: BMC Genomics. 2017 Aug 21;18:638. doi: 10.1186/s12864-017-3996-8 (PMC5563900; doi:10.1186/s12864-017-3996-8)
Supplement: Supplementary file 1 — Reproducibility of ribo-seq and RNA-seq experiments. Figure S2. The number of differentially expressed genes increased as exposure to hypoxia increased. Figure S3. Dynamic translational regulation in ARPE-19 cells upon exposure to hypoxia. Table S1. Overview of ribosome profiling and RNA sequencing data. Table S2. Pathway enrichment of differentially expressed genes. Table S3. Pathway enrichment of differentially translated genes. Table S4. Pathway enrichment of genes with differential translation efficiency. Table S5. Synthesized uORFs/no-uORFs sequence. (DOCX 1503 kb) [file 12864_2017_3996_MOESM1_ESM.docx]

# Ribosome Profiling Reveals Translational Regulation of

# Mammalian Cells in Response to Hypoxic Stress

# Supplemental Figures and Tables

**
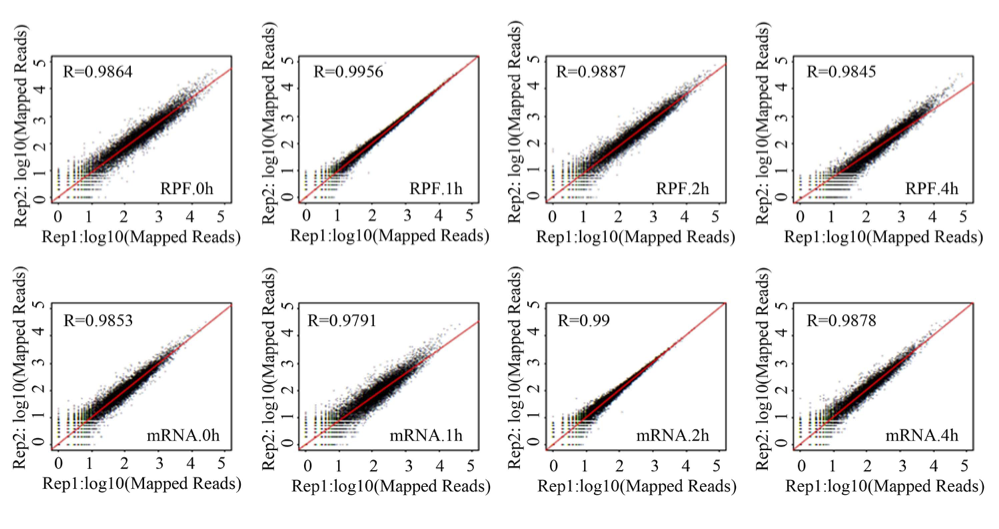
**

**Figure S1 Reproducibility of ribo-seq and RNA-seq experiments**

Two independent experiments of ribo-seq paired with RNA-seq were performed after ARPE-19 cells were exposed to hypoxic stress for 0h, 1h, 2h and 4h. Pearson correlation coefficients of ribo-seq or RNA-seq were calculated based on mapping to human genome reads.

**
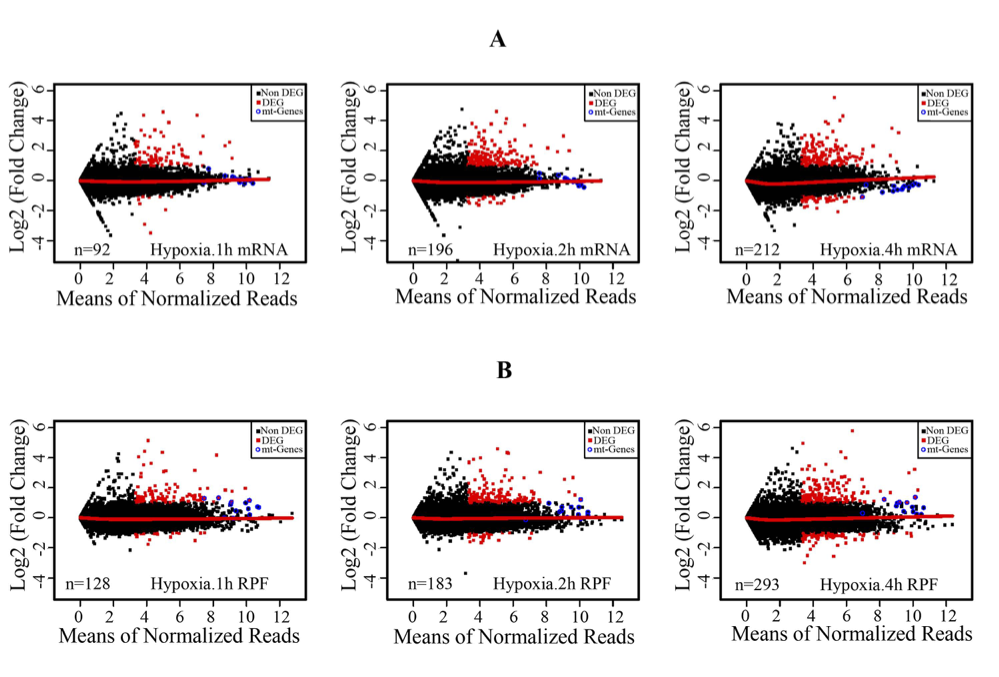
Figure S2 The number of differentially expressed genes increased as exposure to hypox****ia increased**

Differential gene expression analysis of RNA-seq and Ribo-seq indicated that the number of differentially expressed genes increased in ARPE-19 cells exposed to hypoxic stress prolongation for 1h compared with 4h. Black points indicate no differential expression of genes (Non-DEG). Red points indicate differentially expressed genes (DEG). Blue points indicate mitochondrial genes (mt-genes).


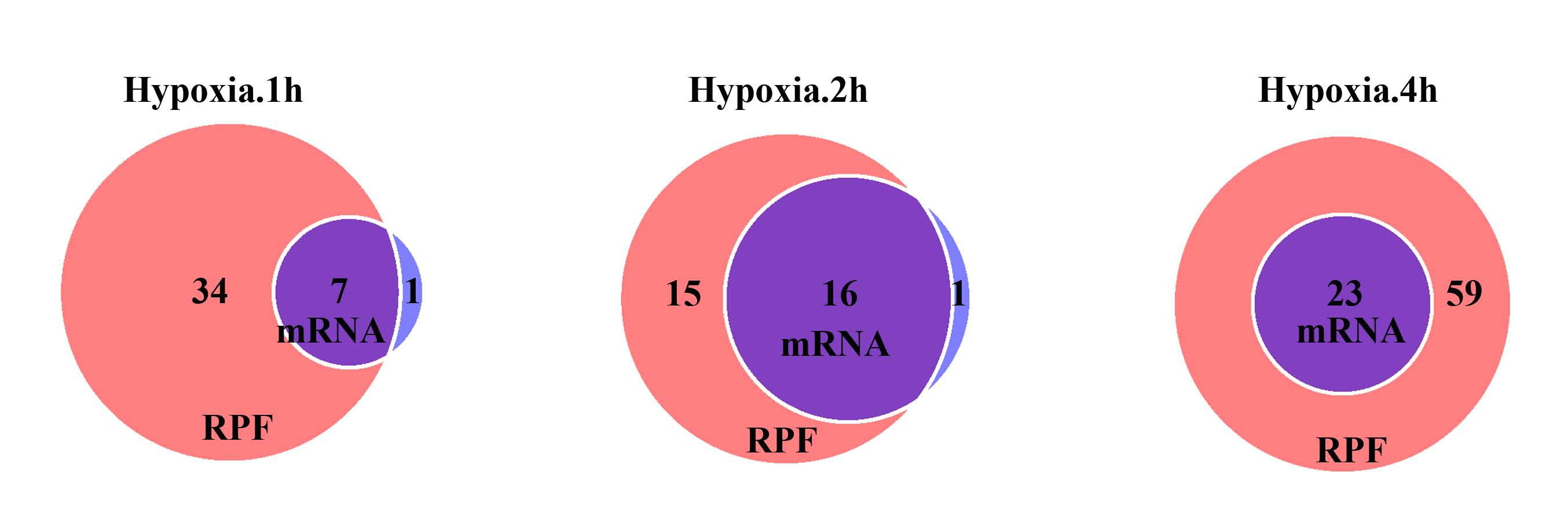


**Figure S3 Dynamic translational regulation in ARPE-19 cells upon exposure to hypoxia**

Venn diagram summarizing differentially expressed genes via mRNA and RPF enrichment of KEGG pathways.

**Table. S1. Overview of ribosome profiling and RNA sequencing data**

| Samples | Raw reads | Adapter removed reads | tRNA/rRNA removed reads | Mapped reads | uniquely aligned reads |
| --- | --- | --- | --- | --- | --- |
| mRNA_0h_1st | 26,581,612 | 23,316,638 | 21,870,727 | 11,786,191 | 9,501,729 |
| mRNA_1h_1st | 20,241,895 | 19,851,646 | 18,720,867 | 8,387,017 | 6,550,860 |
| mRNA_2h_1st | 18,162,777 | 16,956,165 | 15,912,512 | 7,125,131 | 5,695,698 |
| mRNA_4h_1st | 24,405,664 | 23,418,007 | 22,362,221 | 11,484,215 | 9,353,615 |
| RPF_0h_1st | 22,040,020 | 21,283,269 | 17,949,319 | 11,736,795 | 6,923,129 |
| RPF_1h_1st | 32,474,528 | 30,974,649 | 22,762,805 | 14,411,084 | 10,261,763 |
| RPF_2h_1st | 24,010,227 | 23,094,559 | 19,721,941 | 12,013,228 | 8,783,396 |
| RPF_4h_1st | 8,184,139 | 7,510,707 | 6,778,278 | 4,057,996 | 2,976,847 |
| mRNA_0h_2nd | 20,478,484 | 19,862,913 | 18,987,056 | 9,623,915 | 7,604,744 |
| mRNA_1h_2nd | 23,485,675 | 21,719,879 | 21,060,470 | 12,638,246 | 10,486,678 |
| mRNA_2h_2nd | 16,904,652 | 15,737,119 | 15,090,460 | 6,678,110 | 5,458,181 |
| mRNA_4h_2nd | 18,203,179 | 16,840,389 | 16,376,593 | 9,379,941 | 7,884,549 |
| RPF_0h_2nd | 24,251,241 | 22,457,879 | 18,946,181 | 14,737,448 | 11,220,532 |
| RPF_1h_2nd | 28,918,503 | 27,059,522 | 19,872,925 | 12,913,451 | 9,292,243 |
| RPF_2h_2nd | 32,038,642 | 20,922,539 | 17,943,662 | 13,668,893 | 10,223,889 |
| RPF_4h_2nd | 24,454,558 | 19,569,098 | 16,577,476 | 13,061,651 | 9,339,801 |

**Table. S2. Pathway enrichment of differentially expressed genes**

| **1h vs 0h** | | **2 vs 0h** | | **4h vs 0h** | |
| --- | --- | --- | --- | --- | --- |
| KEGG Pathway | Adjust p-value (1h) | KEGG Pathway | Adjust p-value (2h) | KEGG Pathway | Adjust p-value (4h) |
| MAPK signaling pathway | 0.0000 | MAPK signaling pathway | 0.0000 | Apoptosis | 0.0000 |
| p53 signaling pathway | 0.0000 | p53 signaling pathway | 0.0000 | MAPK signaling pathway | 0.0000 |
| Cell cycle | 0.0396 | Cell cycle | 0.0000 | p53 signaling pathway | 0.0000 |
| Toll-like receptor signaling pathway | 0.0396 | Small cell lung cancer | 0.004 | Prostate cancer | 0.0000 |
| Colorectal cancer | 0.0792 | Toll-like receptor signaling pathway | 0.004 | Small cell lung cancer | 0.0000 |
| Epithelial cell signaling in Helicobacter pylori infection | 0.0792 | Epithelial cell signaling in Helicobacter pylori infection | 0.0057 | Aminoacyl-tRNA biosynthesis | 0.0033 |
| B cell receptor signaling pathway | 0.0792 | Prostate cancer | 0.0057 | TGF-beta signaling pathway | 0.0141 |
| Circadian rhythm | 0.0792 | Bladder cancer | 0.0099 | Adipocytokine signaling pathway | 0.0149 |
|  |  | Circadian rhythm | 0.0198 | Bladder cancer | 0.0149 |
|  |  | Apoptosis | 0.0238 | Cell cycle | 0.0149 |
|  |  | DNA replication | 0.0548 | Epithelial cell signaling in Helicobacter pylori infection | 0.0149 |
|  |  | Chronic myeloid leukemia | 0.0548 | Toll-like receptor signaling pathway | 0.0149 |
|  |  | TGF-beta signaling pathway | 0.0548 | Chronic myeloid leukemia | 0.0168 |
|  |  | Pancreatic cancer | 0.082 | Pancreatic cancer | 0.0170 |
|  |  | Focal adhesion | 0.0842 | Cytokine-cytokine receptor interaction | 0.0251 |
|  |  | Aminoacyl-tRNA biosynthesis | 0.0842 | Prion disease | 0.0334 |
|  |  | Colorectal cancer | 0.0978 | Ascorbate and aldarate metabolism | 0.0419 |
|  |  |  |  | Glycine, serine and threonine metabolism | 0.0451 |
|  |  |  |  | One carbon pool by folate | 0.0531 |
|  |  |  |  | Limonene and pinene degradation | 0.0604 |
|  |  |  |  | Cysteine metabolism | 0.0688 |
|  |  |  |  | Methionine metabolism | 0.0918 |
|  |  |  |  | Acute myeloid leukemia | 0.0999 |

**Table. S3. Pathway enrichment of differentially translated genes**

| **1h vs 0h** | | **2h vs 0h** | | **4h vs 0h** | |
| --- | --- | --- | --- | --- | --- |
| KEGG Pathway | Adjust p-value (1h) | KEGG Pathway | Adjust p-value (2h) | KEGG Pathway | Adjust p-value (4h) |
| MAPK signaling pathway | 0.0000 | MAPK signaling pathway | 0.0000 | MAPK signaling pathway | 0.0000 |
| Cell cycle | 0.0000 | Cell cycle | 0.0000 | Cell cycle | 0.0000 |
| Ribosome | 0.0000 | Ribosome | 0.0000 | p53 signaling pathway | 0.0000 |
| p53 signaling pathway | 0.0033 | p53 signaling pathway | 0.0000 | Bladder cancer | 0.0000 |
| Bladder cancer | 0.0033 | Bladder cancer | 0.0000 | Epithelial cell signaling in Helicobacter pylori infection | 0.0000 |
| Colorectal cancer | 0.0033 | Epithelial cell signaling in Helicobacter pylori infection | 0.0000 | Prostate cancer | 0.0000 |
| Epithelial cell signaling in Helicobacter pylori infection | 0.0085 | Prostate cancer | 0.0000 | Apoptosis | 0.0000 |
| Toll-like receptor signaling pathway | 0.0099 | Apoptosis | 0.0000 | Small cell lung cancer | 0.0000 |
| Small cell lung cancer | 0.0178 | Small cell lung cancer | 0.0020 | DNA replication | 0.0000 |
| Chronic myeloid leukemia | 0.0178 | DNA replication | 0.0020 | Chronic myeloid leukemia | 0.0000 |
| Ubiquitin mediated proteolysis | 0.0180 | Chronic myeloid leukemia | 0.0083 | Colorectal cancer | 0.0000 |
| ErbB signaling pathway | 0.0330 | Pyrimidine metabolism | 0.0083 | Pancreatic cancer | 0.0000 |
| Prostate cancer | 0.0411 | Colorectal cancer | 0.0172 | Systemic lupus erythematosus | 0.0000 |
| DNA replication | 0.0424 | Renal cell carcinoma | 0.0172 | Ubiquitin mediated proteolysis | 0.0014 |
| Glycan structures - biosynthesis 1 | 0.0436 | Prion disease | 0.0172 | Renal cell carcinoma | 0.0026 |
| B cell receptor signaling pathway | 0.0439 | Pancreatic cancer | 0.0173 | Prion disease | 0.0035 |
| Renal cell carcinoma | 0.0439 | Ubiquitin mediated proteolysis | 0.0198 | Base excision repair | 0.0035 |
| Vibrio cholerae infection | 0.0439 | Focal adhesion | 0.0198 | Glycine, serine and threonine metabolism | 0.0044 |
| Nucleotide excision repair | 0.0439 | Toll-like receptor signaling pathway | 0.0292 | Phosphatidylinositol signaling system | 0.0050 |
| GnRH signaling pathway | 0.0439 | Nucleotide excision repair | 0.0327 | Inositol phosphate metabolism | 0.0050 |
| Pyrimidine metabolism | 0.0439 | Glycan structures - biosynthesis 1 | 0.0380 | Acute myeloid leukemia | 0.0057 |
| Systemic lupus erythematosus | 0.0439 | mTOR signaling pathway | 0.0380 | Melanoma | 0.0072 |
| Oxidative phosphorylation | 0.0439 | Base excision repair | 0.0380 | Axon guidance | 0.0083 |
| Wnt signaling pathway | 0.0539 | Axon guidance | 0.0380 | TGF-beta signaling pathway | 0.0083 |
| mTOR signaling pathway | 0.0539 | Melanoma | 0.0396 | Nitrogen metabolism | 0.0087 |
| Base excision repair | 0.0602 | TGF-beta signaling pathway | 0.0442 | Cysteine metabolism | 0.0110 |
| Notch signaling pathway | 0.0645 | Systemic lupus erythematosus | 0.0462 | Aminoacyl-tRNA biosynthesis | 0.0110 |
| Pancreatic cancer | 0.0686 | Regulation of actin cytoskeleton | 0.0587 | Toll-like receptor signaling pathway | 0.0116 |
| Axon guidance | 0.0696 | Chondroitin sulfate biosynthesis | 0.0690 | Nucleotide excision repair | 0.0116 |
| TGF-beta signaling pathway | 0.0733 | Circadian rhythm | 0.0772 | Neurodegenerative Diseases | 0.0125 |
| Apoptosis | 0.0856 | Mismatch repair | 0.0792 | Pyrimidine metabolism | 0.0128 |
| N-Glycan biosynthesis | 0.0860 |  |  | Glioma | 0.0130 |
| Glioma | 0.0919 |  |  | Focal adhesion | 0.0150 |
| Adherens junction | 0.0919 |  |  | Nucleotide sugars metabolism | 0.0151 |
| Biosynthesis of steroids | 0.0919 |  |  | Ribosome | 0.0164 |
| Thyroid cancer | 0.0919 |  |  | B cell receptor signaling pathway | 0.0198 |
| Glycosylphosphatidylinositol(GPI)-anchor biosynthesis | 0.0920 |  |  | Methionine metabolism | 0.0198 |
| Prion disease | 0.0947 |  |  | Alanine and aspartate metabolism | 0.0214 |
| Focal adhesion | 0.0947 |  |  | Dentatorubropallidoluysian atrophy (DRPLA) | 0.0274 |
| Insulin signaling pathway | 0.0947 |  |  | Glutamate metabolism | 0.0282 |
| RNA polymerase | 0.0947 |  |  | Pathogenic Escherichia coli infection - EHEC | 0.0302 |
|  |  |  |  | Pathogenic Escherichia coli infection - EPEC | 0.0302 |
|  |  |  |  | Oxidative phosphorylation | 0.0309 |
|  |  |  |  | Non-small cell lung cancer | 0.0315 |
|  |  |  |  | mTOR signaling pathway | 0.0331 |
|  |  |  |  | Adherens junction | 0.0331 |
|  |  |  |  | Adipocytokine signaling pathway | 0.0334 |
|  |  |  |  | Aminosugars metabolism | 0.0334 |
|  |  |  |  | Ascorbate and aldarate metabolism | 0.0396 |
|  |  |  |  | T cell receptor signaling pathway | 0.0444 |
|  |  |  |  | Fructose and mannose metabolism | 0.0516 |
|  |  |  |  | Homologous recombination | 0.0518 |
|  |  |  |  | Chondroitin sulfate biosynthesis | 0.0524 |
|  |  |  |  | Benzoate degradation via CoA ligation | 0.0524 |
|  |  |  |  | Vitamin B6 metabolism | 0.0565 |
|  |  |  |  | Glycan structures - biosynthesis 1 | 0.0598 |
|  |  |  |  | Limonene and pinene degradation | 0.0601 |
|  |  |  |  | One carbon pool by folate | 0.0601 |
|  |  |  |  | Cytokine-cytokine receptor interaction | 0.0607 |
|  |  |  |  | Lysine degradation | 0.0607 |
|  |  |  |  | Regulation of actin cytoskeleton | 0.0640 |
|  |  |  |  | Folate biosynthesis | 0.0640 |
|  |  |  |  | Terpenoid biosynthesis | 0.0640 |
|  |  |  |  | Vibrio cholerae infection | 0.0640 |
|  |  |  |  | Urea cycle and metabolism of amino groups | 0.0667 |
|  |  |  |  | Glycerolipid metabolism | 0.0723 |
|  |  |  |  | Circadian rhythm | 0.0736 |
|  |  |  |  | Glyoxylate and dicarboxylate metabolism | 0.0751 |
|  |  |  |  | RNA polymerase | 0.0772 |
|  |  |  |  | Purine metabolism | 0.0772 |
|  |  |  |  | N-Glycan biosynthesis | 0.0772 |
|  |  |  |  | Butanoate metabolism | 0.0853 |
|  |  |  |  | VEGF signaling pathway | 0.0871 |
|  |  |  |  | Mismatch repair | 0.0886 |
|  |  |  |  | Galactose metabolism | 0.0886 |
|  |  |  |  | Phenylalanine, tyrosine and tryptophan biosynthesis | 0.0886 |
|  |  |  |  | Streptomycin biosynthesis | 0.0887 |
|  |  |  |  | Tryptophan metabolism | 0.0896 |
|  |  |  |  | Valine, leucine and isoleucine degradation | 0.0896 |
|  |  |  |  | Caprolactam degradation | 0.0896 |
|  |  |  |  | PPAR signaling pathway | 0.0914 |
|  |  |  |  | Wnt signaling pathway | 0.0988 |

**Table. S4. Pathway enrichment of genes with differential translation efficiency**

| **Time** | **KEGG Pathway** | **Adjust p-value** |
| --- | --- | --- |
| **1h vs 0h** | ribosome | 9.55E-05 |
|  | chondroitin sulfate biosynthesis | 0.03255 |
|  | ecm-receptor interaction | 0.094289 |
|  | glycan structures - biosynthesis 1 | 0.094289 |
| **2h vs 0h** | ribosome | 2.02E-15 |
| **4h vs 0h** | Neuroactive ligand-receptor interaction | 0 |
|  | Cell Communication | 0.0665 |

**Table. S5. Synthesized uORFs/no-uORFs sequence**

| uORFs/no-uORFs | Sequence (5'-3') |
| --- | --- |
| EPO_uORF | ATGAGGGCCCCCGGTGTGGTCACCCGGCGCGCCCCAGGTCGCTGA |
| EPO_no-uORF | CTGAGGGCCCCCGGTGTGGTCACCCGGCGCGCCCCAGGTCGCTGA |
| NAIP_uORF | ATGACAGTGAGCTAACTGGTGGCTGGGATCCTTTAGACAACTTCAGGATGGGGGCTATCCCCTGAAAGACTAAGGCATGATTAGATGCCTAAGTGATGGCACCTCCATTAAAAAATAAACCACAGGTTTGGAGAGCTTTCGGTTTGGTTAACCCCAACCACATACCAAGAAGGCGATGCACCTCAAACTGCATGA |
| NAIP_ no-uORF | CTGACAGTGAGCTAACTGGTGGCTGGGATCCTTTAGACAACTTCAGGATGGGGGCTATCCCCTGAAAGACTAAGGCATGATTAGCTGCCTAAGTGATGGCACCTCCATTAAAAAATAAACCACAGGTTTGGAGAGCTTTCGGTTTGGTTAACCCCAACCACATACCAAGAAGGCGATGCACCTCAAACTGCATGA |
| PNPLA7_uORF | ATGACAGACACTTCTCAAAAGACAGCTTTTCTTCCTGGAGAACAGACTTTTTCAGCAGGATTTTCCTTTCAGTGA |
| PNPLA7_ no-uORF | CTGACAGACACTTCTCAAAAGACAGCTTTTCTTCCTGGAGAACAGACTTTTTCAGCAGGATTTTCCTTTCAGTGA |
| SMPDL3B_uORF | ATGCCTGCCTCCTCGGGCCAGCCCAGATCATACCCTGCTGGGCAAAGGAGGAAGAGCCAGAGGATCCAGACGCCTTGGAGGACTTGGAACACCTGTAACAGGACAAGGAGTTCTGCTCAGGCACGTGGCCACAGAAAACTACTTAGGAAGCCTGTGGTGAGAACAACAACAGTGCCTGAGAATCCCACGGCTCTGGGGAAGTGA |
| SMPDL3B_no-uORF | CTGCCTGCCTCCTCGGGCCAGCCCAGATCATACCCTGCTGGGCAAAGGAGGAAGAGCCAGAGGATCCAGACGCCTTGGAGGACTTGGAACACCTGTAACAGGACAAGGAGTTCTGCTCAGGCACGTGGCCACAGAAAACTACTTAGGAAGCCTGTGGTGAGAACAACAACAGTGCCTGAGAATCCCACGGCTCTGGGGAAGTGA |
